# Unilateral Mooren’s ulcer in a patient with bilateral partial optic nerve atrophy - a case report

**DOI:** 10.22336/rjo.2022.64

**Published:** 2022

**Authors:** Mioara-Laura Macovei, Adelina-Maria Neacșu

**Affiliations:** * Department of Ophthalmology, “Dr. Carol Davila” Central Military Emergency University Hospital, Bucharest, Romania

**Keywords:** Mooren’s ulcer, peripheral ulcerative keratitis, partial nerve atrophy

## Abstract

**Objective:** The aim of this report is to present the diagnostic and therapeutic approach in a case of long standing peripheral ulcerative keratitis of a patient with already decreased visual acuity due to preexistent optic nerve partial atrophy.

**Case presentation:** A 58-year-old male patient presented to our clinic with unilateral pain, photophobia, tearing and decreased visual acuity in the right eye. The symptomatology began 4 months prior to the presentation, after trauma with a vegetative corneal foreign body, for which he received treatment in another Ophthalmology Service. After every specific cause of peripheral ulcerative keratitis was excluded, using extensive laboratory testing, the diagnosis of unilateral Mooren’s ulcer in the right eye was established and the topical steroid treatment was initiated. A very good response to the treatment was observed, with complete resolution of the affliction.

**Discussions:** Mooren’s ulcer represents an idiopathic form of peripheral ulcerative keratitis. The diagnostic approach in this type of cases consists in the exclusion of infectious etiology (to safely use topical steroids) and of specific autoimmune etiologies. As the evolution of this pathology is severe with frequent complications, the treatment must be initiated as soon as possible.

**Conclusions:** Despite being a rare diagnosis, Mooren’s ulcer must be considered in cases in which clinical and paraclinical presentation is highly suggestive. A rapid therapeutic approach can offer good results despite the usual severe evolution of this disease.

## Introduction

Mooren’s ulcer represents an idiopathic, autoimmune, corneal ulceration with chronic evolution, located in the peripheral cornea. It is a rare disease, one study conducted in China approximating its incidence to be 0.03% [**1**]. 

The most common symptoms are: pain (that can be severe), photophobia, tearing and decreased visual acuity [**2**]. The clinical presentation is with a crescent-shaped peripheral ulceration that involves the superficial stroma, situated 2-3 mm from the limbus, without affecting the Descemet’s membrane or the sclera [**3**]. The ulceration distinctively has a central undermined leading edge and vascularization originating from the conjunctiva that may involve the ulcer’s bed, but that does not advance past this edge [**4**]. It is characterized by a circumferential progression followed by a central spread [**5**].

Two clinical types of Mooren’s ulcer have been described. The first type, usually unilateral, affecting the elderly population, has a slower, less severe progression and a good response to medical treatment. The second type is characterized by a bilateral presentation, is commonly described in younger African population and unfolds with increased severity and a poor response to therapy [**4**]. The etiology is unknown, however, trauma, ocular surgery or exposure to parasitic infection may precipitate the affliction [**2**]. A strong association with hepatitis C is also described, in which case, Interferon therapy may represent a solution [**6**]. Studies suggest a possible link between Mooren’s ulcer and certain HLA alleles. One study described a higher prevalence of HLA-DR17 and HLA-DQ2 in the affected patients compared to the control population [**7**]. Astigmatism represents a frequent complication of the disease, alongside perforation, which usually develops after a minor trauma [**3**]. Since topical steroids represent the main therapy, complications of their long-term use, such as cataract, glaucoma or secondary bacterial infection, may occur [**4**].

## Case presentation

A 58-year-old Caucasian male presented to our clinic for unilateral pain, tearing, photophobia and decreased vision, affecting the right eye (OD). The patient reported that the onset of the symptoms occurred 4 months earlier, following ocular trauma with a vegetal corneal foreign body. After the foreign body was removed in another Ophthalmology Service, the patient was treated with topical broad-spectrum antibiotics and lubricants in the affected eye, without any improvement. The diagnosis was reconsidered one month prior to the presentation to metaherpetic keratitis, therefore, when we first encountered the case, the patient was following a topical treatment in the right eye with antivirals and lubricants. 

The past ocular history revealed partial optic nerve atrophy affecting both eyes (right eye - due to an episode of non-arteritic anterior ischemic optic neuropathy diagnosed 7 years back, left eye - due to contusive ocular trauma reported 5 years before presentation). The family medical history was unremarkable. 

**Fig. 1:**
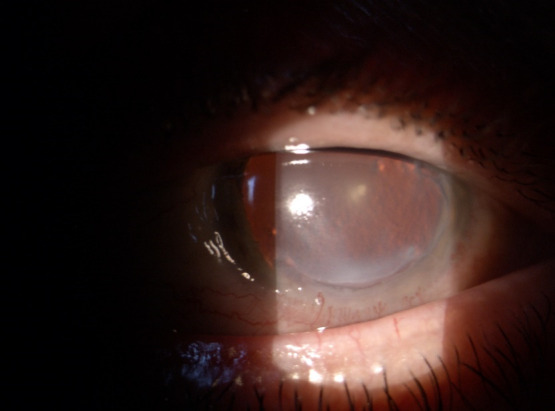
OD slit lamp image

At the clinical ocular exam, the best corrected visual acuity was 0.1 in the right eye and 0.2 in left eye, while the intraocular noncontact pressure was 20 mmHg in both eyes. The slit lamp exam of the right eye revealed conjunctival injection, a peripheral, crescent-shaped corneal ulcer situated 2 mm from the limbus, extended on approximately 180° inferiorly and nasally (between 7:00 and 1:00 o’clock) (**Fig. 1**). 

**Fig. 2:**
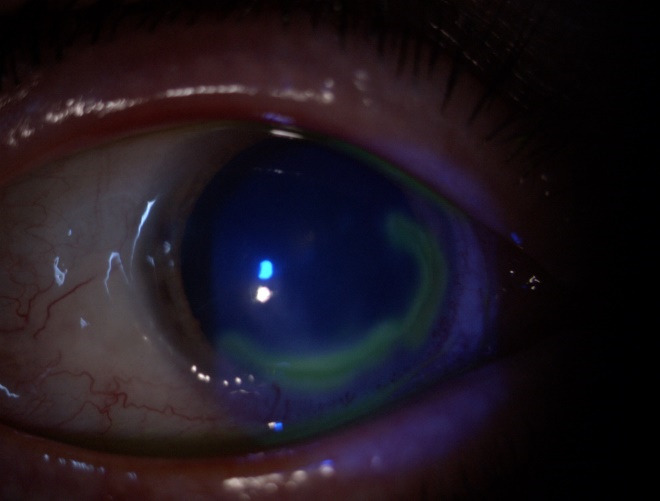
OD: fluorescein stain + cobalt blue

The defect involving the epithelium and the superficial stroma is better illustrated after fluorescein staining (**Fig. 2**). 

Conjunctival blood vessels invading the peripheral margin of the ulcer were documented. The corneal sensitivity was normal. At the examination of the left eye’s anterior pole, no pathological findings were noted.

**Fig. 3:**
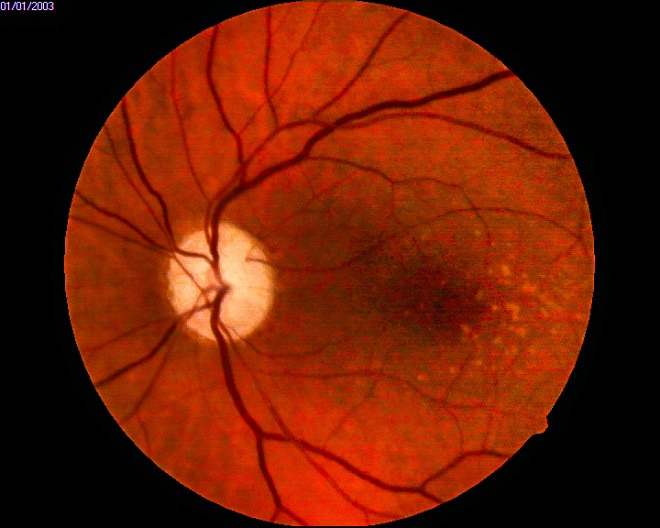
OD - fundus image

**Fig. 4:**
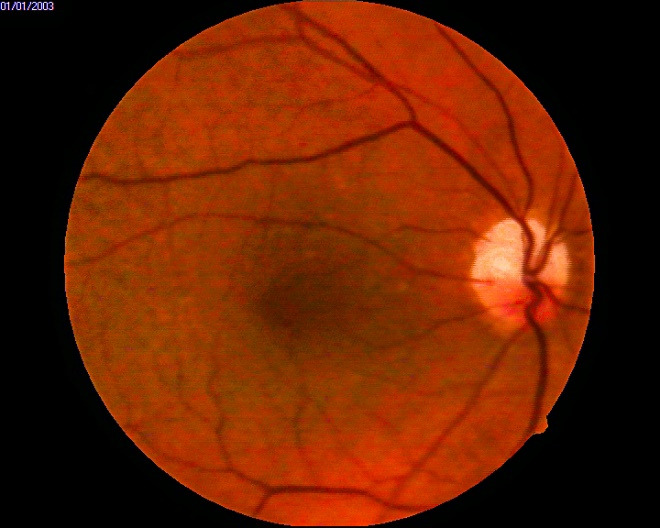
OS - fundus image

Fundus examination of both eyes revealed a pale neuroretinal rim (superior two thirds in the right eye (**Fig. 3**), entirely in the left eye (**Fig. 4**)), an absent foveal reflex, numerous macular, small, round, white-yellow subretinal deposits, located around the fovea (highly suggestive of macular hard drusen).

**Fig. 5:**
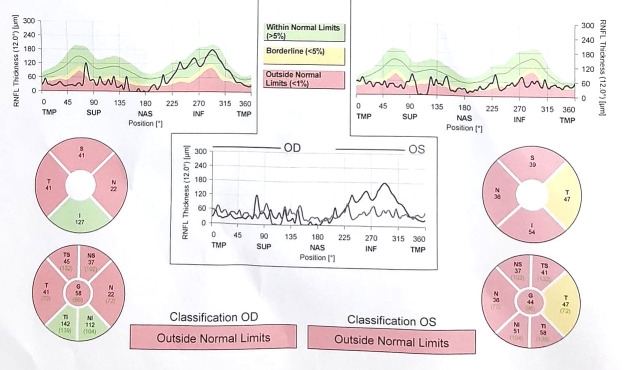
OU: RNFL Exam Report

The RNFL exam showed decreased thickness in the superior, temporal and nasal quadrants in the right eye, while in the left eye RNFL thickness was decreased in the superior, nasal and inferior quadrants, with borderline values for the temporal quadrant (**Fig. 5**). The visual field examination revealed a dense inferior altitudinal defect in the right eye and severe visual field constriction in the left eye.

An extensive laboratory evaluation was initiated. The blood investigations including complete blood count, biochemistry, coagulation tests, liver and kidney function, glycemia, electrolytes and inflammatory markers were within normal limits. Hepatitis C markers (anti-HCV antibodies, viral ARN) and syphilis screening test (RPR) were negative. Further immunological tests were also performed: rheumatoid factor, anti-CCP antibodies, antinuclear antibodies, pANCA, cANCA, anti-Ro and anti-La antibodies were within normal range. Urinary sediment analysis revealed no modifications. A superficial corneal swab was collected for several microbiological cultures and the results proved to be negative.

**Fig. 6:**
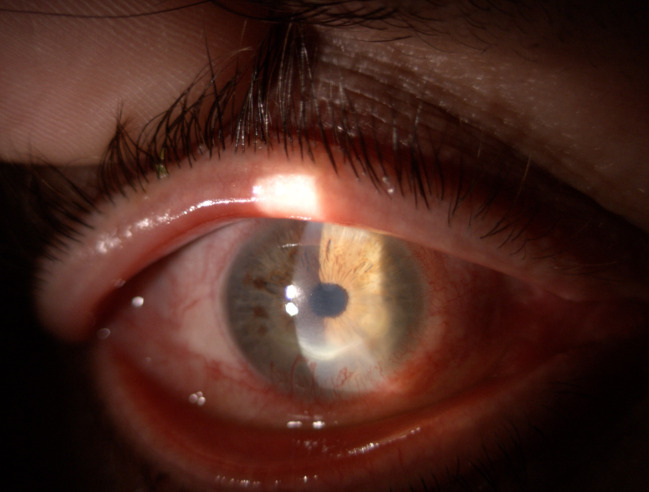
OD: slit lamp exam - central migration

**Fig. 7:**
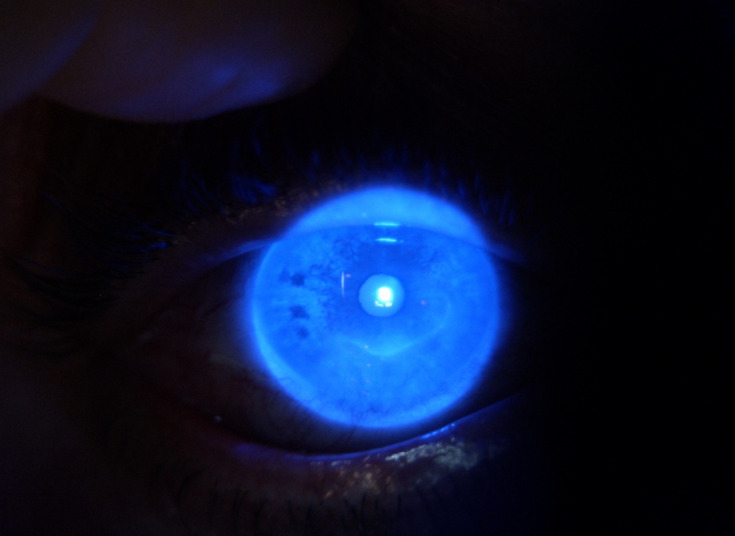
OD: fluorescein stain + cobalt blue

At the initial presentation, while waiting for the laboratory results to establish a diagnosis, the patient was kept under observation and received topical treatment for the right eye with wide-spectrum antibiotics (5 times daily) and preservative-free lubricants (5 times daily), and oral treatment with retinal and optic nerve supplements. An aggravation of the corneal lesion was noted in this period, with central migration of the ulcer, further advancement of the corneal blood vessels close to the ulceration’s edge (**Fig. 6**) and increased astigmatism (+2.75/-2.50/85°). The base of the lesion stained with fluorescein (**Fig. 7**).

Noting this evolution and after eliminating all the specific causes of peripheral ulcerative keratitis (PUK), a final principal diagnosis of right unilateral Mooren’s ulcer was established, while secondary diagnosis for both eyes were: partial optic atrophy, macular drusen, simple hyperopic astigmatism and presbyopia. Treatment with topical steroids (5 times daily) was initiated in the right eye [**8**].

A topical combination of a Carbonic Anhydrase Inhibitor and a Beta-blocker was administered twice daily to avoid intraocular pressure increase under steroid treatment (given the existing optic nerve damage). Prophylactic topical antibiotics and lubricants were used until the closure of the epithelial defect. Retinal and optic nerve oral supplements were continued permanently.

**Fig. 8:**
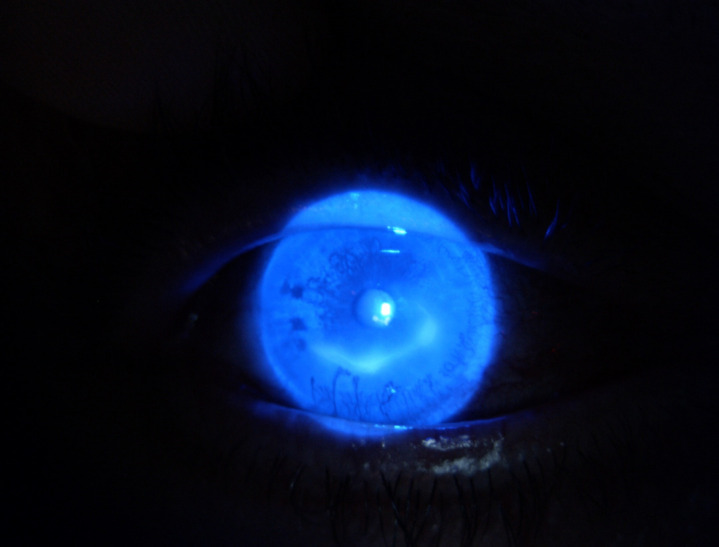
OD: after 5 days of topical steroids

**Fig. 9:**
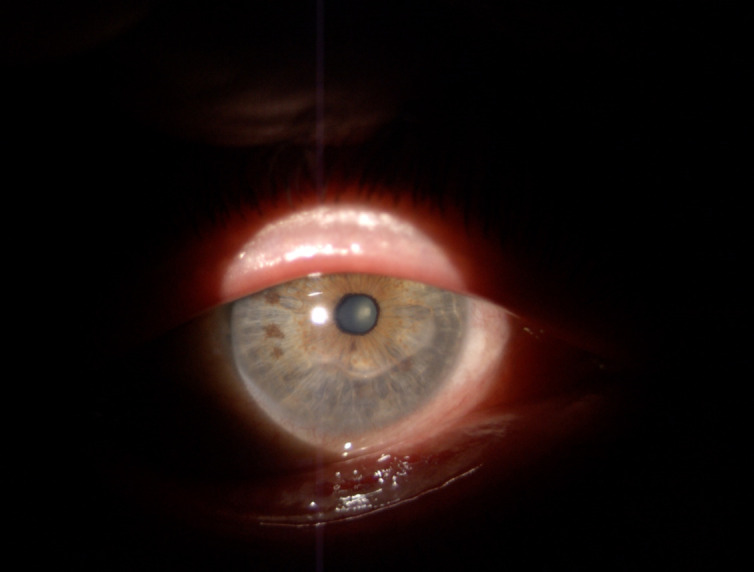
OD: after 1 month of treatment

A 5 days follow-up showed an improved visual acuity (0.3 with best correction), complete regression of the corneal blood vessels and a shallow ulcer that stained with fluorescein only centrally (**Fig. 8**). Also, a decreased corneal astigmatism was noted (+1.75/ -1.75/ 83°). The topical treatment with steroids was tapered within the next weeks until cessation. At the 1-month follow-up, the visual acuity in the right eye was 0.5 with best correction (the same visual acuity as before the ulcer’s onset), noncontact tonometry showed 18 mmHg intraocular pressure and corneal astigmatism was diminished (+1.25/ -1.25/ 78°). The complete closing of the epithelial defect and regression of corneal blood vessels were observed (**Fig. 9**).

## Discussion

Mooren’s ulcer represents an idiopathic condition, the diagnosis being one of exclusion. The first diagnosis that was ruled out by the negative corneal cultures was bacterial keratitis. Staphylococcal marginal keratitis was improbable in the absence of blepharitis and given the long case history. Fungal keratitis could appear in a case with vegetative corneal foreign body and without antifungal prophylaxis, but the clinical presentation and the negative cultures denied this diagnosis. Herpetic marginal keratitis and metaherpetic keratitis were excluded in the presence of normal corneal sensitivity. Once the infections hypothesis was ruled out, the next stage of the differential diagnosis was with systemic autoimmune diseases. Granulomatosis with polyangiitis, Eosinophilic granulomatosis with polyangiitis, Rheumatoid arthritis, Systemic lupus erythematosus and Sjögren syndrome were excluded by the absence of their specific markers at the laboratory evaluation.

Despite the spectacular response to topical treatment, the ulcer’s relapse remained the main concern in this case, therefore, long-term follow-up, including after steroid cessation, was compulsory. In case of relapse, alternative treatments such as topical ciclosporin 2%, conjunctival resection, lamellar keratectomy, systemic immunosuppression [**5**], must be taken into consideration.

Even though the corneal lesion presented a complete remission, the long-term visual prognosis was worsened by the presence of bilateral optic nerve atrophy.

## Conclusions

In conclusion, although a very rare diagnosis, Mooren’s ulcer must be considered in a long standing peripheral ulcerative keratitis once the infectious hypothesis is eliminated. The aim of this case report was to illustrate the diagnostic and therapeutic approach in a unilateral Mooren’s ulcer that responded well to therapy, in the case of a patient in whom the presence of bilateral partial optic nerve atrophy aggravated the condition.

**Conflict of Interest Statement**

The authors state no conflict of interest.

**Informed Consent and Human and Animal Rights statement**

An informed consent has been obtained from the patient included in the case report.

**Authorization for the use of human subjects**

Ethical approval: The research related to human use complies with all the relevant national regulations, institutional policies, it is in accordance with the tenets of the Helsinki Declaration and has been approved by the review board of the Ophthalmology Department of “Dr. Carol Davila” Central Military Emergency University Hospital, Bucharest, Romania.

**Acknowledgements**

None.

**Sources of Funding**

None.

**Disclosures**

None.

**Contribution**

Both authors contributed equally to this article.
